# Measuring Gait Velocity and Stride Length with an Ultrawide Bandwidth Local Positioning System and an Inertial Measurement Unit

**DOI:** 10.3390/s21092896

**Published:** 2021-04-21

**Authors:** Pratham Singh, Michael Esposito, Zach Barrons, Christian A. Clermont, John Wannop, Darren Stefanyshyn

**Affiliations:** 1 Biomedical Engineering Graduate Program, Schulich School of Engineering, University of Calgary, Calgary, AB T2N 1N4, Canada; pratham.singh@ucalgary.ca (P.S.); michael.esposito@ucalgary.ca (M.E.); 2 Faculty of Kinesiology, University of Calgary, Calgary, AB T2N 1N4, Canada; zachary.barrons@ucalgary.ca (Z.B.); Christian.clermont@ucalgary.ca (C.A.C.); b.wannop@ucalgary.ca (J.W.)

**Keywords:** local positioning system, Bland–Altman, motion analysis, gait

## Abstract

One possible modality to profile gait speed and stride length includes using wearable technologies. Wearable technology using global positioning system (GPS) receivers may not be a feasible means to measure gait speed. An alternative may include a local positioning system (LPS). Considering that LPS wearables are not good at determining gait events such as heel strikes, applying sensor fusion with an inertial measurement unit (IMU) may be beneficial. Speed and stride length determined from an ultrawide bandwidth LPS equipped with an IMU were compared to video motion capture (i.e., the “gold standard”) as the criterion standard. Ninety participants performed trials at three self-selected walk, run and sprint speeds. After processing location, speed and acceleration data from the measurement systems, speed between the last five meters and stride length in the last stride of the trial were analyzed. Small biases and strong positive intraclass correlations (0.9–1.0) between the LPS and “the gold standard” were found. The significance of the study is that the LPS can be a valid method to determine speed and stride length. Variability of speed and stride length can be reduced when exploring data processing methods that can better extract speed and stride length measurements.

## 1. Introduction

Coaches and athletes are interested in profiling speed since the ability to produce high speeds is considered an important quality for performance [1]. Athlete rehabilitation may also benefit from accurately profiling speed [2]. Additional biomechanical data, such as stride length and stride frequency, are also beneficial for coaches and athletes because they provide more in-depth analyses of gait biomechanics. For example, speed, a key indicator of performance, is the product of stride length and stride frequency. Previously, coaches and training staff needed expensive lab equipment such as video motion capture (MOCAP) to obtain accurate information. Data collection was also time consuming because video data typically required post processing and analysis for biomechanical assessments. Wearable sensors are now more advanced and allow laboratory quality data to be easily collected by athletes in a setting that is more representative of a field of play. They have minimal constraints on the athlete and result in performances more like their typical gait patterns [3]. The processing system in wearable sensor technology can also provide real-time data. Therefore, it would be beneficial for coaches and training staff to use wearable technology provided they generate accurate position and velocity data from their athlete in order to help improve race performance.

Runners’ positions can be obtained with the help of Global Positioning System (GPS)-based wearable technologies and runners’ accelerations can be obtained with an inertial measurement unit (IMU) [4,5]. However, there are limitations with GPS and an IMU. GPS technology is limited as previous research has shown that it may be difficult to operate a GPS-based wearable in some outdoor settings [6]. GPS has low position accuracy when the satellite visibility is reduced. There are also limitations with an IMU. An IMU is extremely sensitive to drift errors (i.e., an offset in the accelerometer signal output) [7,8]. As a result, an IMU is not very good at determining position on its own, especially over long periods of time due to drift. A drift error will result in positional errors and reduce the accuracy of spatial parameters such as stride length and temporal parameters such as running speed that are important for examining performance [9]. Therefore, more precise sensors are needed in order to track subtle movements.

A more precise system may include an ultrawide bandwidth Local Positioning System (LPS) [10]. Ultrawide bandwidth technology is a radio frequency signal that operates in a bandwidth equal to or greater than 500 MHz [11]. Most off-the-shelf GPS systems only have a sampling rate of 5 Hz or 10 Hz [4], and over 75% of runners at an official road race event use GPS-based watches [12], which typically have a sampling rate of 1 Hz [13]. If the sampling rate is lower than 10 Hz, precision and accuracy may be reduced, as there may be critical position data points not being measured. Jennings et al. [14] failed to reach acceptable validity (10.4–12.7% standard estimate of error) for distance measures when subjects were walking and running while using a 1 Hz GPS. Furthermore, an LPS is like a GPS in that there is a sensor and a hub used to collect and store data. In a GPS, the satellite used to collect data can be quite distant. However, unlike a GPS, in an LPS, the source of the radio wave emitter is in close proximity to the user. This attribute of an LPS is gaining popularity due to the hub allowing collection in both an outdoor and indoor environment [15,16]. The LPS hub can also collect and store position information in direct line of sight to the user. Therefore, LPS is very good at determining position [17]. However, obtaining gait events such as step count and heel strike using accelerations from an LPS may lack efficacy. Stevens et al. [18] determined the accuracy of the peak accelerations to be limited when measuring subjects performing soccer-specific movements with a radio-frequency-based LPS.

It may be beneficial to get peak vertical acceleration from an IMU rather than the LPS to determine heel strike. The peak vertical accelerations would be obtained after double differentiating the position data when using an LPS. An IMU would directly measure peak vertical acceleration from the heel strike and, as a result, the peaks would be more pronounced when using an IMU and the chance of an error would be reduced. Therefore, a potential solution to mitigate measurement errors could be a sensor fusion method where an IMU could first characterize gait events [19], such as heel strike to heel strike (stride), and then accurate position data during the stride could be obtained from the ultrawide bandwidth LPS.

The assessment of bias is important because large differences can be detrimental in determining performance, especially for athletes. In a sporting context, even if the bias is small from the LPS, the variability needs to be taken into account by the coach. Possible inaccurate speed and stride length measurements could result in a coach prescribing inappropriate training regimens for the athlete, while the athlete could be spending their time on techniques that do not require improving. A sport scientist who is looking at spatial-temporal trends would not be able to manage training loads as accurately as possible for an athlete. An opportunity still exists for ultrawide bandwidth-based LPSs equipped with IMU sensors to provide more precise and comprehensive sets of data to coaches, athletes and sport scientists through the development of new processing algorithms.

An IMU sensor equipped with a radio frequency emitter operating in the ultrawide bandwidth are complimentary and pairing them may produce a better outcome [20]. The output from the sensor fusion would, therefore, be stride length. This fusion resolves issues seen by the IMU and eliminates drift error by directly collecting position data. To combine both accurate gait event data from the IMU and accurate position data from the LPS, sensor fusion is required. Sensor fusion increases accuracy and certainty when data is being observed [21]. Therefore, the purpose of the project was to:

Obtain walking, running and sprinting speed and stride length from an LPS operating in the ultrawide bandwidth equipped with an IMU and compare to a criterion standard (i.e., the “gold standard”) such as MOCAP.

## 2. Materials and Methods

Ninety participants (Males: 59, Females: 31, Age: 27.6 years ± 5.1 (mean ± SD), Mass: 70.7 kg ± 10.6, Height: 173.8 cm ± 8.7) free of any physical condition that prevented them from running were recruited to participate in the study. Prior to participation, participants were provided with a written and verbal explanation of the protocol. Written and informed consent was obtained from all participants. This study was approved by the University of Calgary Conjoint Health Research Ethics Board (REB17-0614).

**Figure 1:**
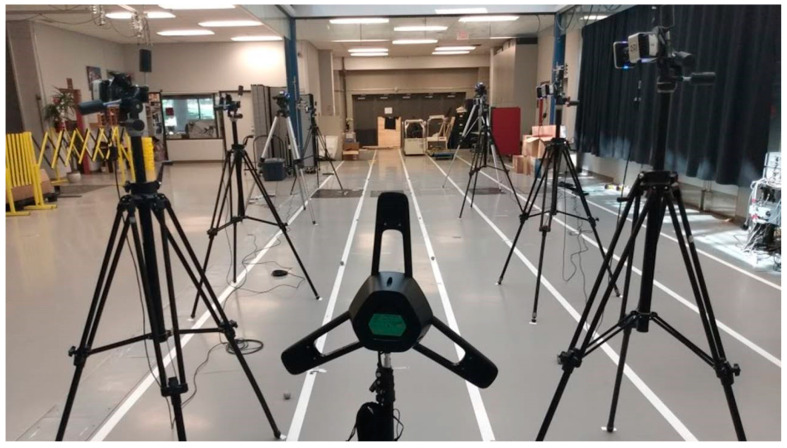
The setup included eight motion capture cameras surrounding the ten-meter-long running lane. The LPS hub was placed on a tripod, 2 m behind the starting position.

As seen in Figure 1, the testing session was conducted along a ten-meter running lane. The LPS (XCO Tech Inc., Penticton, BC, Canada) consisted of a single hub (nominal measuring range: 100 m) and an IMU sensor (Model: MPU9250, Accelerometer range: ±8 g) equipped with a radio frequency emitter operating in the ultrawide bandwidth (central frequency: 4 GHz; bandwidth: 500 MHz). The hub was positioned two meters behind the participant’s starting position at a height of one meter. The IMU sensor was placed in a stitched pocket of a belt comfortably secured over the sacrum of the participant, near the participant’s center of mass, a position previously shown to produce a reliable measurement of acceleration patterns in walking and running [5]. The IMU was positioned such that the *x*-axis was oriented in the anterior–posterior direction, *y*-axis was oriented in the medial–lateral direction and *z*-axis was oriented in the superior–inferior direction along a conventional x, y, z coordinate system. The LPS and the IMU data were collected at 100 Hz. Kinematic data were recorded using retroreflective markers and eight MOCAP cameras (Vicon, Oxford, UK). MOCAP was also collected at 100 Hz. The measurement error in MOCAP was below 0.2 mm (i.e., difference between 2D images of the markers and the 3D reconstruction of those markers was less than 0.2 mm). A retroreflective marker was placed on each heel and on the belt containing the IMU.

**Figure 2:**
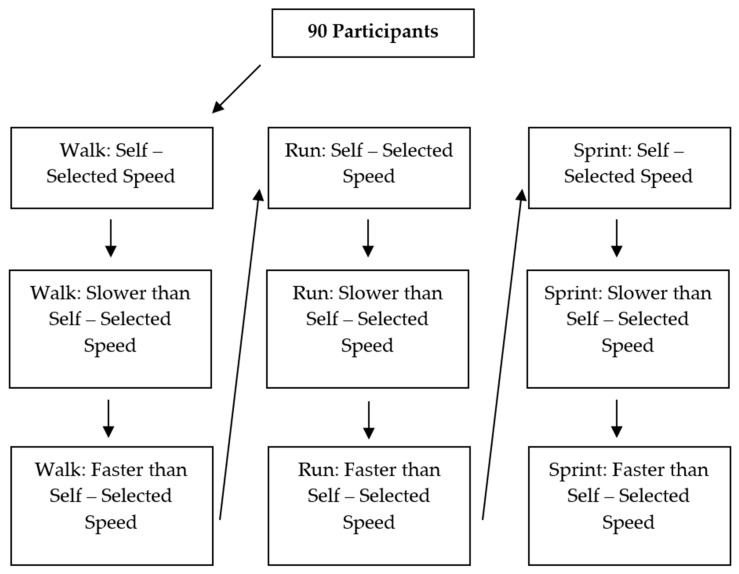
A flowchart highlighting the order of the protocol performed.

Participants were permitted to walk or run the length of the running lane prior to data collection in order to familiarize themselves with the laboratory setup. After which, three trials were performed of three speeds in the following order: walk, run and sprint (Figure 2). The first trial of each condition was at a self-selected speed. The participant was told to perform the second trial slower than their self-selected speed (any speed slower than their self-selected speed). The participant was then told to perform the third trial faster than their self-selected speed (any speed faster than their self-selected speed). To obtain a quick estimate of the speed, Brower timing gates (Brower Timing Systems, Draper, UT, USA) were placed eight and ten meters from the starting position on either side of the running lane. Figure 2 shows the order in which the data were collected. As a result, several different speeds were obtained for each condition and a large spectrum of gait speeds were collected for analysis. Due to the presence of metal objects such as tripods and MOCAP cameras in the laboratory environment, reflections of the ultrawide bandwidth radio waves may have interfered with the signal [22]. To check for these types of multi-path events, the standard deviation of the ultrawide bandwidth radio waves was calculated from a six-sample moving box car for the phase difference from each antenna pair; if the standard deviation exceeded 6 mm (i.e., three standard deviations from the mean), the trial was flagged as anomalous (herein after referred to as an “anomaly” or “anomalies”). Speed across the last five meters and stride length across the last stride was analyzed before and after the anomalies were removed.

Speed from the LPS was obtained by finding the change in the *x*-axis position across the last five meters per unit time. Stride segmentation from the LPS was obtained by finding peaks in the superior-inferior direction of the IMU data from the accelerometer and deeming them as heel strikes [23]. Stride length was calculated as the difference in position obtained from the LPS.

After the MOCAP cameras were calibrated, retroreflective markers were placed at the start and end points of the running lane. Retroreflective markers at the start and end point confirmed the start position and end position during data processing. Speed from the MOCAP was calculated by finding the change in the sacrum marker’s position per unit time. Again, the speed was calculated as the change in position across the last five meters per unit time. Stride length from the MOCAP was obtained by finding the difference in the *x*-axis position. This was calculated between the time points of consecutive ipsilateral vertical position minima of a heel marker (i.e., heel strikes). The stride length in the last stride was used for analysis.

Speed across the last five meters was chosen for analysis because the LPS may be used by high level athletes to improve their performance. Sustaining near maximal speeds is strongly related to match play performance [24], while the relative distance displaced per stride has a strong positive relationship to a male sprinter’s strength capacity [25]. The last stride was used in all trials to ensure the participant had reached a steady state in their gait.

MOCAP, acceleration and location data were processed using Vicon Nexus software and MATLAB R2018a (Mathworks Inc., Natick, MA, USA). The MOCAP and LPS signals were aligned using cross correlation. A rotational matrix was employed to reorient the LPS coordinate system to the MOCAP coordinate system. A Kalman filter was used on the LPS position data. Using the Nyquist frequency, a low-pass filter with a cutoff frequency of 50 Hz was used to process the IMU acceleration signal. Bland–Altman plots were created in Microsoft Excel (Microsoft Corporation, Redmond, WA, USA) to compare speed and stride length between the MOCAP and LPS [26]. Bland–Altman plots allow for a visual representation of how well a system performs in comparison to a “gold standard”. The mean bias and average between MOCAP and LPS, were obtained from the Bland–Altman plots. A root mean square error (RMSE) value was also calculated for speed and stride length, with and without anomalies. A two-way mixed, average measure Intraclass Correlation Coefficient (ICC) was calculated using IBM SPSS Statistics Version 26 (IBM Corporation, Armonk, NY, USA).

## 3. Results

**Figure 3:**
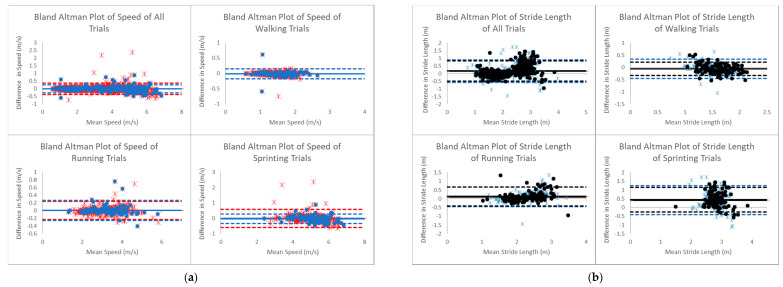
(**a**) Bland–Altman plot of speed comparing difference (MOCAP minus ultrawide bandwidth LPS) and mean. Red stars and red dashed lines (±1.96 SD from the bias line) represent a dataset where anomalies are not removed. Dark blue dots and dark blue dashed lines represent a dataset where anomalies are removed. (**b**) Bland–Altman plot of stride length comparing difference (MOCAP minus ultrawide bandwidth LPS/IMU) and mean. Light blue stars and light blue dashed lines represent a dataset where anomalies are not removed. Black dots and black dashed lines represent a dataset where anomalies are removed.

Out of 810 trials, there were 358 trials (126 walking trials, 114 running trials and 118 sprinting trials) without anomalies. As shown in Figure 3, for all trials, the bias in the Bland–Altman Plot of speed from MOCAP and LPS was 4.99 × 10^−3^ m/s, the upper limit of agreement (+1.96 SD) was 0.37 m/s and the lower limit of agreement (−1.96 SD) was −0.38 m/s. The RMSE of speed was 0.19 m/s. The ICC of speed from MOCAP and LPS was 1.00. Once the anomalies were removed, the bias in the Bland–Altman Plot of speed from MOCAP and LPS was −0.01 m/s, the upper limit of agreement was 0.25 m/s and the lower limit of agreement was −0.27 m/s. The RMSE of speed was 0.15 m/s. The ICC of speed from MOCAP and LPS was 1.00. The bias in the Bland–Altman Plot of stride length was 0.15 m, the upper limit of agreement was 0.89 m and the lower limit of agreement was −0.58 m. The RMSE of stride length was 0.41 m. The ICC of stride length was 0.90. Once the anomalies were removed, the bias in the Bland–Altman Plot of stride length was 0.17 m, the upper limit of agreement was 0.83 m and the lower limit of agreement was −0.49 m. The RMSE of stride length was 0.41 m. The ICC of stride length was 0.90. The Bland–Altman plots comparing MOCAP and LPS showed a large distribution. The speed mean between MOCAP and LPS ranged from 0.58 m/s to 6.84 m/s while the difference between MOCAP and LPS ranged from −0.75 m/s to 2.38 m/s. The stride length mean ranged between 0.73 m to 3.97 m while the difference between MOCAP and LPS ranged between −1.44 m to 1.73 m. Once the anomalies were removed, the speed mean between MOCAP and LPS ranged from 0.58 m/s to 6.84 m/s while the difference between MOCAP and LPS ranged from −0.59 m/s to 0.89 m/s. Again, once the anomalies were removed, the stride length mean ranged between 1.03 m to 3.86 m while the difference between MOCAP and LPS ranged between −0.95 m to 1.44 m.

Table 1 shows the key results from the Bland–Altman plots comparing the speed in the anterior-posterior direction.

## 4. Discussion

The purpose of this project was to calculate and compare speed and stride length using position data from an LPS equipped with an IMU to MOCAP, the “gold standard”, and to determine whether agreement varied across different forms of gait (walk, run and sprint). Bland–Altman plots were applied in order to be able to make speed and stride length comparisons. Based on the Bland–Altman plots, the study suggests a single hub ultrawide bandwidth LPS equipped with an IMU can be a valid method to determine speed and stride length when appropriate data processing methods are used.

Although a novel, single-hub-based ultrawide bandwidth LPS system was used in this study, the assessment of measuring speed agrees with previous research on multi-hub ultrawide bandwidth systems. Such systems were shown to provide valid measurements for applications in indoor sport settings [27] and can be used in time-motion sport studies [28]. Luteberget, Spencer and Gilgien’s [28] statistical analysis is comparable to the analysis done in this study and reported a similar bias (0.05 m/s ± 0.14 m/s). Serpiello et al. [27] do caution against the use of such systems for determining precise measurements of peak speeds. Further research on the processing of the LPS data could lead to improvements of accuracy of instantaneous and peak speeds. In general, another limitation of the LPS includes a nominal measuring range that can be small [29]. Nevertheless, the results of this study agree with other studies that show ultra-wideband systems can provide more accurate measures of speed than GPS wearable solutions [16]. The standard deviation of speed in the sprinting trials in this study was 0.16 m/s while Munoz-Lopez et al. [30] reported a standard deviation of 0.77 m/s in a 5 Hz GPS unit. This may have implications for athletic talent scouts because, for younger athletes, the speed achieved during a five-meter sprint is a key indicator in soccer talent identification with differences of just 0.15 m/s between drafted and non-drafted players [31].

The Bland–Altman analysis reported 95% limits of agreement and a bias for speed and stride length. However, there are no guidelines on interpreting the limits of agreement and bias, but rather, every Bland–Altman analysis must be considered with reference to the range of the data [32]. When comparing the Bland–Altman plots of speed and stride length between LPS and MOCAP, outliers in speed and stride length plots and the range of differences can be visualized. Looking at all of the speed trials comparing MOCAP and LPS, the mean bias is low but there is a considerable amount of variation during running and sprinting trials, making it difficult to rely on individual measurements. Looking at all the stride lengths between MOCAP and LPS, an even larger amount of variation is present in the bias as well as a non-zero mean. However, after removing anomalous trials using a simple filter, the variability is reduced. Since ultrawide bandwidth covers such a large frequency spectrum, there may have been interference from reflections off metallic materials or electrical components that operate at a lower frequency within the laboratory environment [22,33]. Future work should explore if the variability is also reduced in regular indoor and outdoor sport settings. In addition, when collecting trials, it may be advantageous to notify coaches, athletes and sport scientists of an anomaly in real time, giving them the opportunity to recollect the trial.

With the data collected in this project, there is a large range of speeds and stride lengths in walking, running and sprinting trials. The results showed the limits of agreement were closer to the bias in walking and running trials than in the sprinting trials. This finding is corroborated by previous research as it was revealed that the typical error of the estimate becomes larger during linear sprints when assessing validity of an LPS in the ultrawide bandwidth [34]. Those findings showed the accuracy of an LPS may be reduced for assessing sprints due to a low sampling frequency of position measurements taken during such brief efforts. Based on previous research exploring the validity of a radio frequency-based LPS during sprints and obtaining high validity correlations, an effective sampling frequency of 330 Hz may be a more appropriate sampling frequency [35]. Nevertheless, 100 Hz was used in this project and better estimates of speeds and stride lengths may be found if higher sampling frequencies are used.

The advantage of the single hub LPS used in this study is that 3D position can be determined without multiple anchors (or hubs) and calibration. However, the system is not any more immune than other radio wave-based positioning systems to reflections that create multi-path interference which in turn degrade position determination accuracy [22]. Although all systems incorporate signal and data processing methods to mitigate these errors, they do not account for all possible reflections. Given that multi-path induced errors are dependent on waves bouncing off of nearby surfaces (e.g., walls, the ground and equipment) relative to the hub location, it is possible that a second hub placed at a different location would not be impacted by the same level of interference at any given time. Such a two-hub system could choose the position solution from the hub that has the least amount of interference at that time and, therefore, report the most accurate determination of position of the two hubs. This may be more inconvenient and time-consuming for coaches and sport scientists who do not want to spend too much time on setting up equipment and away from their athletes. Furthermore, while a MOCAP system costs approximately $100,000 and a multi-hub system costs approximately $50,000, the LPS using a single hub costs approximately $5000. Future research should investigate the improvement of single hub multi-path processing and also explore the use of additional LPS hubs to find a balance between user burden and improving the accuracy of speed and stride length measurements.

Please note that this project does have its limitations. As mentioned, a greater sampling rate may be beneficial. The low sampling rate used may have also changed the width of the limits of agreement. Furthermore, considering that the anterior-posterior position was considered when measuring speed, future work should explore medial-lateral and superior-inferior directions as it may add more information to running technique regarding, for example, postural sway and vertical oscillation. Future work should also explore the data collection environment and determine the exact source of the interference causing anomalies in the ultra-wideband radio waves.

## 5. Conclusions

In this project, speed and stride length obtained from LPS with synchronized IMU data and MOCAP were compared using a Bland–Altman analysis. Strong correlations were found in the speeds reported by the systems, with a low mean bias. Variation in the bias was low during walking trials and increased for running and sprinting trials. Substantial, but weaker correlations were seen with stride length and substantial differences in bias were seen. Further data processing was able to identify anomalies in the LPS data, allowing for the removal of anomalous trials. Other data processing methods should also be used to explore which pertinent features from a gait cycle might further improve speed and stride length measurements from an LPS with embedded IMU data operating in the ultrawide radio frequency band.

## Figures and Tables

**Table 1:** Key results pertaining to the Bland–Altman plots comparing speed.

All Trials			
Anomalies removed		Anomalies not removed	
Bias (m/s)	−0.01	Bias (m/s)	4.99 × 10^−3^
St. Dev (m/s)	0.13	St. Dev (m/s)	0.19
Lower LOA (m/s)	−0.27	Lower LOA (m/s)	−0.38
Upper LOA (m/s)	0.25	Upper LOA (m/s)	0.37
Mean Range (m/s)	0.58–6.84	Mean Range (m/s)	0.58–6.84
Number of Data Points below Lower LOA	14	Number of Data Points below Lower LOA	16
Number of Data Points above Upper LOA	11	Number of Data Points above Upper LOA	16
Walk			
Bias (m/s)	−0.01	Bias (m/s)	−0.01
St. Dev (m/s)	0.08	St. Dev (m/s)	0.08
Lower LOA (m/s)	−0.18	Lower LOA (m/s)	−0.18
Upper LOA (m/s)	0.15	Upper LOA (m/s)	0.15
Mean Range (m/s)	0.58–2.65	Mean Range (m/s)	0.58–2.65
Number of Data Points below Lower LOA	1	Number of Data Points below Lower LOA	3
Number of Data Points above Upper LOA	1	Number of Data Points above Upper LOA	2
Run			
Bias (m/s)	0.01	Bias (m/s)	4.16 × 10^−3^
St. Dev (m/s)	0.13	St. Dev (m/s)	0.12
Lower LOA (m/s)	−0.25	Lower LOA (m/s)	−0.23
Upper LOA (m/s)	0.27	Upper LOA (m/s)	0.24
Mean Range (m/s)	1.31–5.81	Mean Range (m/s)	1.31–5.83
Number of Data Points below Lower LOA	1	Number of Data Points below Lower LOA	6
Number of Data Points above Upper LOA	3	Number of Data Points above Upper LOA	6
Sprint			
Bias (m/s)	−0.03	Bias (m/s)	−0.01
St. Dev (m/s)	0.16	St. Dev (m/s)	0.30
Lower LOA (m/s)	−0.33	Lower LOA (m/s)	−0.59
Upper LOA (m/s)	0.28	Upper LOA (m/s)	0.58
Mean Range (m/s)	2.42–6.84	Mean Range (m/s)	2.42–6.84
Number of Data Points below Lower LOA	8	Number of Data Points below Lower LOA	2
Number of Data Points above Upper LOA	7	Number of Data Points above Upper LOA	7

## Data Availability

Study did not report any data.
